# New Permutationally Invariant Polynomial Potential Energy Surfaces for H_5_O_2_^+^ with Fast Analytical Gradients Calculated Using Reverse Differentiation

**DOI:** 10.1021/acs.jpca.5c07198

**Published:** 2026-01-07

**Authors:** Saikiran Kotaru, Chen Qu, Paul L. Houston, Qi Yu, Riccardo Conte, Apurba Nandi, Joel M. Bowman

**Affiliations:** † Department of Chemistry and Cherry L. Emerson Center for Scientific Computation, 1371 Emory University, Atlanta, Georgia 30322, United States; ‡ Independent Researcher, Toronto, Ontario M9B0E3, Canada; § Department of Chemistry and Chemical Biology, 138309 Cornell University, Ithaca, New York 14853, United States; ∥ Department of Chemistry, Fudan University, Shanghai 200438, P. R. China; ⊥ Dipartimento di Chimica, 9304 Università Degli Studi di Milano, via Golgi 19, 20133 Milano, Italy; # Department of Physics and Materials Science, University of Luxembourg, L-1511 Luxembourg City, Luxembourg

## Abstract

Given the central importance of the protonated water dimer to the study of the hydrated proton, we report new fits to the previous CCSD­(T) data set of Huang, Braams, and Bowman (HBB) that are more precise and, unlike HBB, provide fast gradients. The new fits, like the HBB one, are based on linear regression with permutationally invariant polynomials (PIPs). The fast gradients are provided via reverse differentiation. They cost roughly just three times the cost for an energy call and are roughly 20 times faster than the HBB numerical gradients. The two new PESs are fits to the original HBB data sets up to roughly 60,000 and to 110,000 cm^–1^. Comparisons to the CCSD­(T) benchmarks and to the HBB results for stationary points and Diffusion Monte Carlo ZPEs are reported and show good agreement.

## Graphical abstract

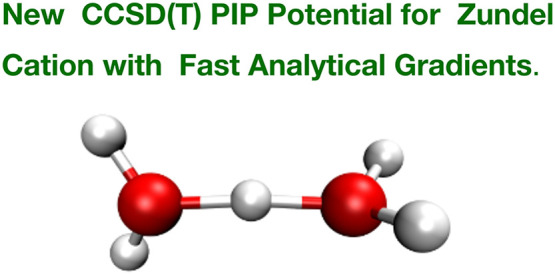

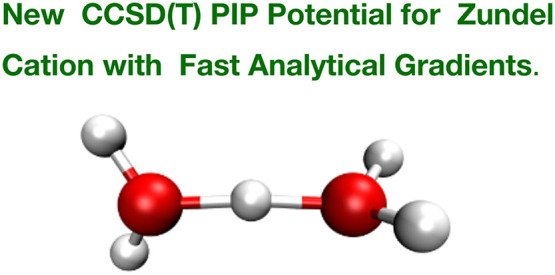

## Introduction

The Zundel cation, H_5_O_2_^+^, is central to our understanding of the hydrated proton. As such, it has received widespread attention both theoretically and experimentally.^1–19^ A systematic study of the dynamical behavior of Zundel contributes to a better understanding of the proton transfer process in bulk water, which is fundamentally significant in chemistry and biology.

The first high-level CCSD­(T)-based, full dimensional potential and MP2-based dipole moment surfaces (DMSs) for H_5_O_2_^+^ were reported in 2005 using permutationally invariant polynomial (PIP) regression.^5^ Significant extensions of that PES and DMS followed roughly a decade later by Yu and Bowman.^20^ Prior to that work, pioneering work describing an MP2-based-PES for the hydrated proton was reported in 1998.^3^ This “OSS” PES was based on elaborating many-component models for the interaction, with numerous linear and nonlinear parameters. These were optimized by nonlinear least-squares fitting of MP2 electronic energies and represented the state of the art at the end of the 20th century. The precision shown in Figure 1 of that paper is certainly below the level routinely seen today and, in particular, shown here. Indeed it was shown in 2005 that the OSS potential for Zundel is not quantitatively accurate.^5^ Multistate empirical valence bond (MS-EVB) potentials for the hydrated proton have also been reported.^21,22^ These potentials are not quantitatively accurate compared to benchmark CCSD­(T) energetics and frequencies for protonated water clusters. In contrast, the many-body PIP PES of Yu and Bowman is in excellent accord with such benchmarks.^15,17,20^ However, it should be noted that this many-body PES was not trained on the liquid hydrated proton, and so it cannot be used directly for such applications. The MB-EVB potentials can be used for these, and a hybrid approach in which snapshots from a molecular dynamics simulation using an MS-EVB potential^23^ were used to obtain vibrational spectra using the many-body PIP PES and DMS has been reported.^24^ Finally, a recent neural network (NN) potential for H_5_O_2_^+^ has been reported,^25^ based on a limited set of CCSD­(T) electronic energies compared to the HBB data set; we discuss this surface below.

**Figure 1:**
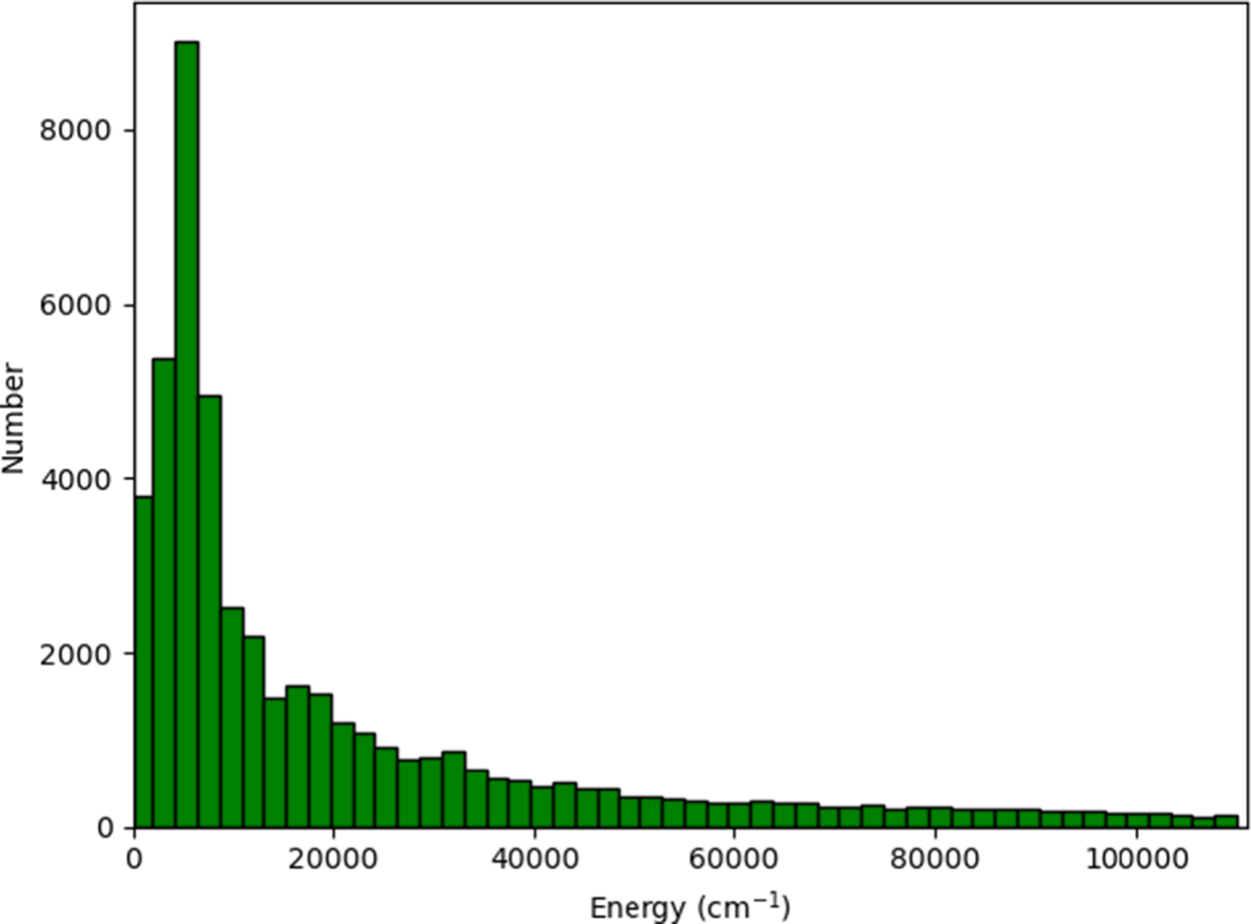
Histogram of CCSD­(*T*) energies relative to the global minimum energy.

The HBB PES does not provide analytical gradients, so obtaining those from finite-difference approximations is computationally slow. Even so, this potential has been used for path integral molecular dynamics of the temperature dependence of the structural fluctuations of H_5_O_2_^+^ and its isotopomer^26^ and “Variational Quantum Monte Carlo with Path Integral Langevin Dynamics” calculations.^27^ Here, we report two new PIP PESs. These are fits to the CCSD­(T)/aVTZ data set of energies used for the 2005 HBB PES. Briefly, the basis sets of PIPS are obtained via MSA software,^28,29^ and they are also postprocessed using Mathematica-based software^30^ to include fast analytical gradients, both those produced by forward and reverse differentiation.

## Methods

### Fitting with PIPs

To begin, recall the expression for the potential is,^28,31^

$$V(y)=\sum_{\alpha =1}^{M}c_{\alpha }\;p_{\alpha }(y)$$ 1

where *c*_α_ are the linear coefficients, *p*_α_ are PIPS, M is the maximum polynomial order, and *y* is the collection of Morse variables. The PESs use a PIP basis of symmetry A_5_B_2_. One new PES uses a PIP basis with a maximum polynomial order of 7, resulting in a size of 8717 polynomials, and is a fit to the HBB data set up to roughly 110,000 cm^–1^; this PES is denoted “PES110 K”. A second new fit uses a basis of 8001 terms and a subset of the HBB data set up to roughly 60,000 cm^–1^; this PES is denoted “PES60K”. The range parameter, *a*, in the Morse variable, exp­(−*r*_*ij*_/*a*), is 3.0 bohr, where *r*_*ij*_ is the internuclear distance between atoms *i* and *j*. Details of these fits are given below.

A histogram of the data set of 48,199 CCSD­(T) energies used in the 2005 paper^5^ is shown in Figure 1. As seen, it extends to very high energies, corresponding to highly distorted structures. Also, there is extensive coverage out to dissociation, namely, O–O distances of 300, 150, 75, 35, 20, 17, 14, 11, 9, 8, 7, 6, 5, and 4 bohr.^5^ The fit was done, as usual, using least-squares without regularization, as described recently.^31^ The energies in hartrees were weighted using the weight *w*(*E*) = Δ/(*E* + Δ), where Δ = 0.1 hartree. Several fits using maximum basis orders of 3–7 were performed using the full range of the data set.

One further fit, along with the 7^th^-order fit, was selected for development. For this further fit, a PIP basis with 8001 polynomials/coefficients was made as described below. While the 7^th^-order basis was fit to all CCSD­(T)/aug-cc-pVTZ geometries shown in Figure 1, the 8001 coefficient fit was fit to a truncated set, one with energies up to a maximum of about 60,000 cm^–1^. The PES60K basis set was obtained by adding basis functions to the 6^th^-order, 2651 coefficient basis using PESPIP software.^30^ Because the product of two PIPs is also a PIP, we can be assured that the permutation symmetry is maintained. To determine which PIPs are most important, we evaluate all possible new PIPs by calculating their values for all geometries in the database and recording the maximum value obtained for each proposed PIP. Recall that the polynomials are Morse variables that decrease with the internuclear distances. Thus, those PIPs with the largest values will correspond to the atom pairs that are closest to one another and should be the most important to keep. We determined these in forming a basis of 8001 PIPs.

Next, we briefly describe the software to use reverse differentiation to produce fast analytical gradients.

### Reverse Differentiation and Fast Analytical Gradients

The last steps for completing the basis set are adding methods for calculating analytical gradients. Figure 2 shows the procedure by using available software. After deciding on the desired permutational symmetry and the polynomial order, the user runs the MSA software^28,29^ (https://github.com/szquchen/MSA-2.0). This software generates a Fortran file, bemsa.f90, which contains the list of monomials and polynomials needed for generating the PIP basis. Optionally, the MSA software can provide analytical gradients. The Mathematica^32^ software developed by our group^30^ (https://github.com/PaulLHouston/PESPIP) reads this file and generates a DuplicatesDeleted.···.f90 file that can be used by further Mathematica software to prune, add, or purify the polynomial basis and to add fast, analytical reverse derivatives. It then outputs a Fortran file for compilation, fitting, and evaluation of the energy and gradients at the desired geometries.

**Figure 2:**
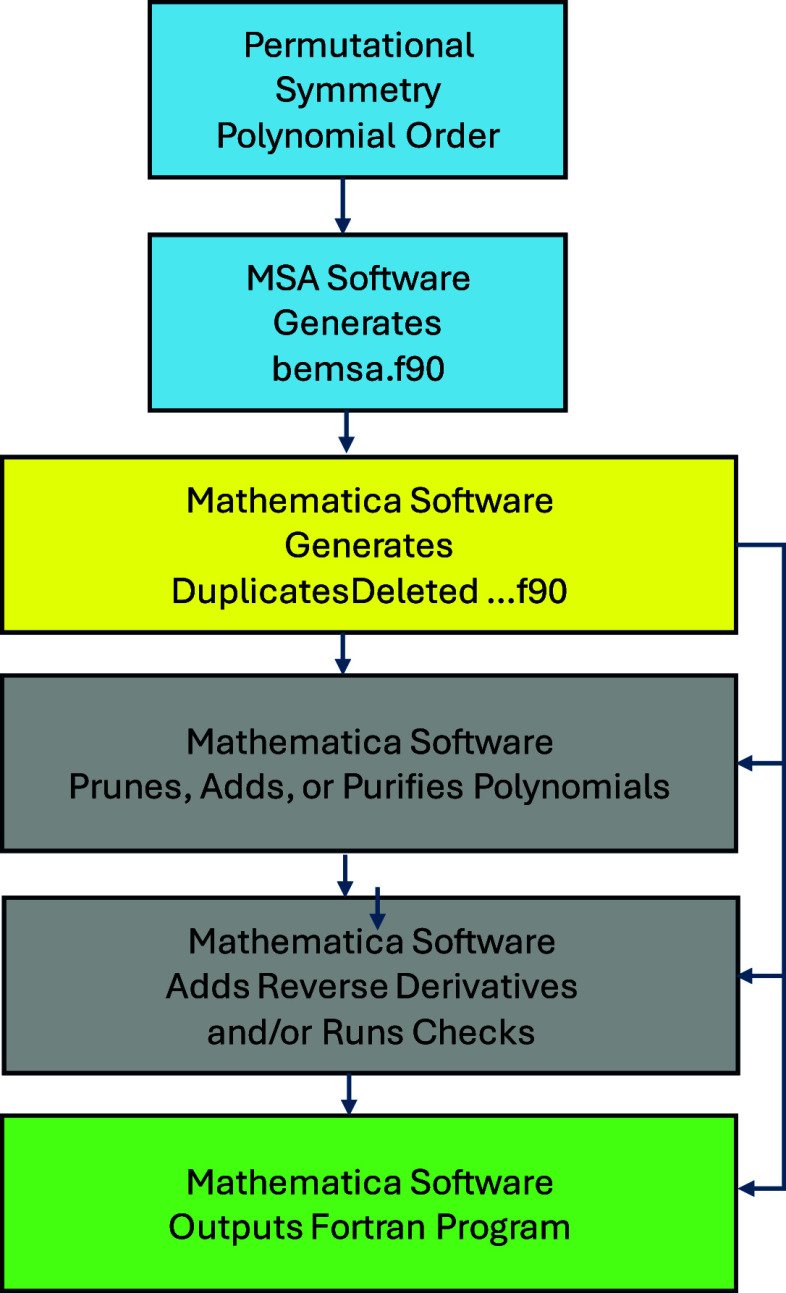
Flowchart for Mathematica software.

The Mathematica software we developed uses symbolic calculations of the formulas needed to modify the basis or to add gradients. It then assembles these formulas into a Fortran program using its text manipulation features. After compilation, the formulas are evaluated in Fortran. The software is described in detail elsewhere,^30^ so that we merely summarize the results concerning the addition of gradients here, emphasizing the difference between “normal analytic” gradients^33,34^ and “reverse” (sometimes called “backward” or “back-propagated”) gradients.^35,36^ The former are obtained by differentiation of both sides of eq 1 to determine how the change in potential depends on the change in *p*_α_ with respect to the Cartesian coordinates. Because this calculation needs to be performed for each coordinate at a unit cost equal to that for determining the energy, the total cost is 3*N* times that for the energy. In contrast, the “gradient by reverse differentiation” method runs a calculation of the energy and then works backward from the energy using partial differentiation to determine *all* the 3*N* gradients in a single pass. The total cost is approximately 3–4 times the cost of the energy and is *independent* of the number of atoms.^30,37^ We used this reverse differentiation method to add fast analytical gradients to both PES60K and PES110 K.

We now clarify an important point: the Mathematica code to generate gradients must be run to generate Fortran output for each permutational symmetry and polynomial order of interest. Thus, there is an overhead on the order of an hour or so to generate the fast derivative method for most problems of interest. Once the basis set and derivative methods have been established, however, they can be run without further change. Also, the basis and reverse derivative code can be used for any molecule with the same permutational symmetry and polynomial order.

Although the Mathematica code is normally run on a computer with a Mathematica license, it can also be run using the freely available Wolfram Engine (https://www.wolfram.com/engine/) with Jupyter Notebook (https://jupyter.org/). The user needs to download and install Wolfram Engine, Jupyter, and the Wolfram Language kernel add-on for Jupyter notebooks (https://github.com/WolframResearch/WolframLanguageForJupyter). Then, the user can (1) download the template from https://github.com/PaulLHouston/PESPIP, (2) copy the code provided in “TemplatesAndExamplesV1.2.nb” into Jupyter Notebook, and (3) run the program to generate the Fortran code with fast reverse derivatives included.

### Diffusion Monte Carlo

Diffusion Monte Carlo (DMC) calculations were performed using the two fits presented in this paper, PES110 K and PES60K. These calculations employ the standard unbiased protocol described in ref 38. Specifically, 10 DMC “trajectories” were run for each PES, and in each DMC trajectory, 30,000 walkers were initiated at the global minimum configuration and were propagated for 55,000 steps, with an imaginary-time step size of 5.0 a.u. The first 5000 steps were used for equilibration, and the reference energies of the remaining 50,000 steps were collected and averaged to calculate the zero-point energy of the molecule. The standard deviations of the ZPEs from the 10 trajectories were calculated to estimate the uncertainty of the DMC calculation.

## Results and Discussion

To begin, we present a mainly pedagogical study of the precision of PIP fits for a variety of sizes of PIP bases and fits to the full data set, which extends to a maximum energy of roughly 110,000 cm^–1^. We also show a result most relevant for the paper, where the data set is restricted to energies up to 60,000 cm^–1^. The former results are shown in Table 1 and graphically in Figure 3. For the largest basis, the unweighted and weighted RMS fitting errors are 36 and 25 cm^–1^, respectively. These are very small and close to the fitting errors reported in 2005.

**Table 1:** Fitting Precision (cm^–1^) for Indicated PIP Bases and Maximum Energy in the Dataset

max order	basis size	max. *E* cm^–1^	RMSE	wRMSE
3	59	110k	5598	3590
4	218	110k	1922	1207
5	772	110k	661	410
6	2651	110k	207	128
7, PES110 K	8717	110k	36	25
6+, PES60K	8001	60k	13	6

**Figure 3:**
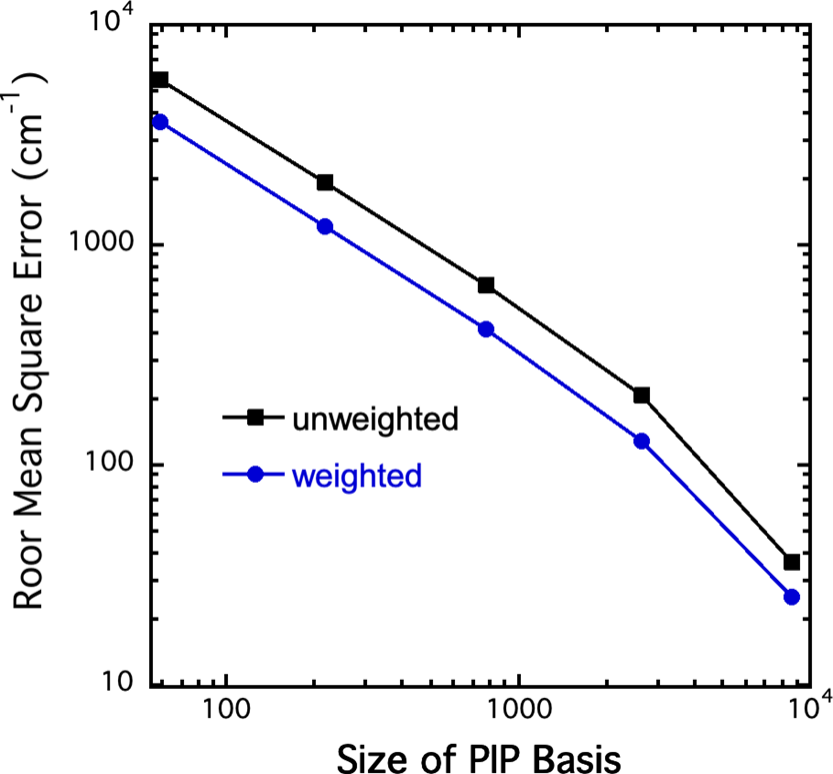
RMSE as a function of basis size for fits to the entire data set of 110,000 energies.

The precision of the fit PES60K using energies up to 60k cm^–1^ is shown in Figure 4. The unweighted rms error is 13.0 cm^–1^, the mean absolute error (MAE) is 6.9 cm^–1^, and the *R* ^2^ coefficient is 0.999999. In addition to normal analytical gradients, this 60k cm^–1^ fit also provides gradients obtained by reverse differentiation^37^ as well as a Hessian calculation based on the reverse differentiation gradients combined with numerical ones. The gradients calculated by reverse differentiation are significantly faster than the normal analytical ones, and both of these are much faster than fully numerical ones.^37^

**Figure 4:**
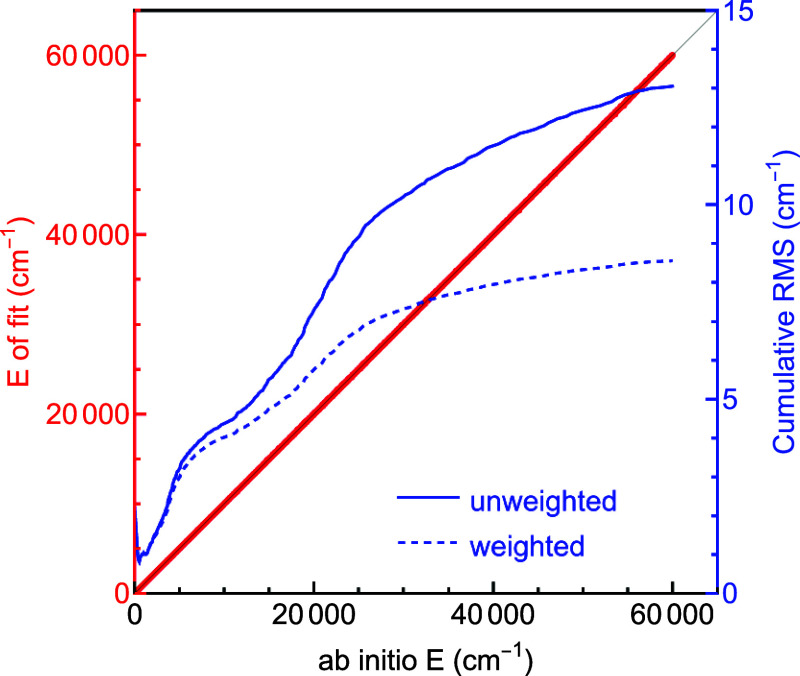
Correlation plot showing the agreement between the ab initio data and the fit, PES60K, to those data using the 8001 coefficient basis.

Timing results using reverse differentiation were measured for the PES60K fit. Using an Intel Core i7–8750H (2.20 GHz) processor, the times for calculating 10,000 energies and 10,000 gradient sets were 1.60 and 3.75 s, respectively.

Normal mode analyses of the PES60K fit are compared to the those using the HBB^5^ PES in Table 2. For the *C*_2_ minimum, the mean absolute error (MAE) for a comparison of the harmonic frequencies is 4.3 cm^–1^, while for the *C*_s_ inv transition state, the MAE is 3.3 cm^–1^. Frequencies and energies for three other low-lying stationary states are also provided. Both surfaces give reasonable agreement with the energies of the CCSD­(T) benchmark. The agreement for the *C*_2h_ trans, *C*_2v_ cis, and *C*_s_ inv transition states is especially noteworthy, as these stationary points are not in the training data set.

**Table 2:**
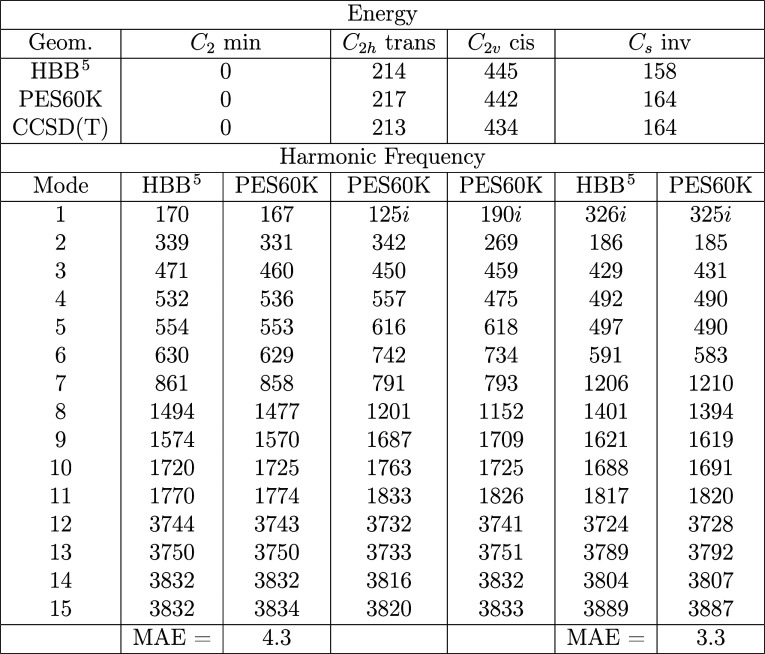
Energies and Harmonic Frequencies (cm^–1^) for Low-lying Stationary Points on the H_5_O_2_^+^ PES60K

DMC calculations were done to locate holes in the PESs. None were found. The ZPE values of H_5_O_2_^+^, using PES110 K and PES60K, are 12402 ± 2 and 12386 ± 2 cm^–1^, respectively. The results are in good agreement with the DMC ZPE of 12395 ± 5 cm^–1^ using the HBB potential.^39^

Figure 5 shows the minimum energy pathway for dissociation/association for H_5_O_2_^+^ ⇌ H_2_O + H_3_O^+^ as a function of the O–O distance on the 8001 coefficient PES, PES60K. This “relaxed” pathway was created by starting with the dissociation products, each in their global minimum configuration, at an O–O distance of 30 bohr and performing a simulated annealing trajectory by decreasing the kinetic energy at each step of the trajectory. The conditions of the trajectory were as follows: an initial kinetic energy of 12 cm^–1^, a time step of 5 a.u., and a loss of 0.01% of the kinetic energy at each time step. In addition, a “brake” was used to prevent excessive kinetic energy from causing the trajectory to leave the minimum energy path; if the kinetic energy exceeded 100 cm^–1^, then it was reduced by 50%. In order to determine the inner wall of the potential cut, a similar trajectory was performed starting at an O–O distance of 3.7 bohr with a time step of 3 au, a loss of 0.005% per step, an initial kinetic energy of 0.1 cm^–1^, and a brake at 6 cm^–1^. Finally, a trajectory starting at 15 bohr with a 4 a.u. step, 0.01% loss, 5 cm^–1^ kinetic energy, and a brake at 6 cm^–1^ was found to agree extremely well with the 30 bohr starting trajectory where they overlapped. The resultant potential energy cut shown in Figure 5 dissociates smoothly and indicates a *D*_e_ of 11,933 cm^–1^, in good agreement with the result on the HBB surface (see ref 5, Figure 5) of 11,924 cm^–1^. In fact, the two PES cuts are nearly indistinguishable. When corrected for zero-point energies, these two numbers are in good agreement with the experimental *D*_0_ values.^5,40^

**Figure 5:**
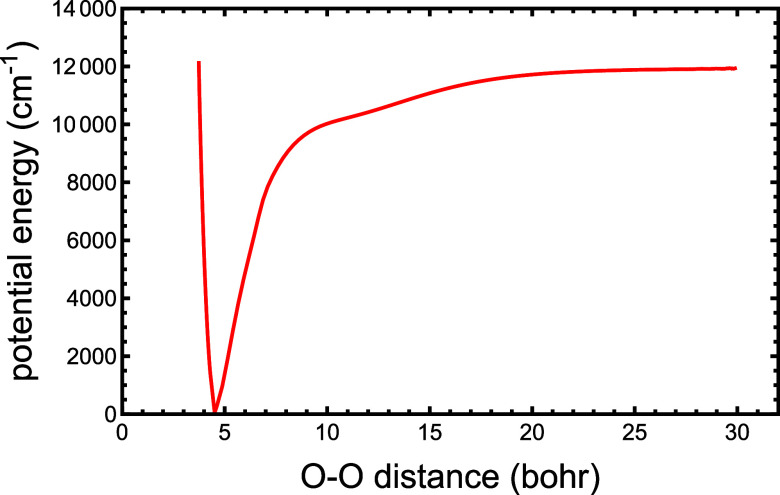
Cut through an 8001 coefficient surface as a function of the O–O distance. At each point along the cut, all of the other coordinates are at their minimum energy. The O–O distance at the minimum is at 4.5124 bohr, and the dissociation energy is 11,933 cm^–1^.

As mentioned in the Introduction, a recent PES for the Zundel cation, called the BBSM PES,^19^ has been reported^25^ that uses an atom-centered, Behler–Parinello neural network fitting approach.^41,42^ It is based on geometries whose energies are calculated using the CCSD­(T*)-F12a/aug-cc-VTZ method, which is better corrected for basis set incompleteness error than the CCSD­(T)/aug-cc-VTZ method used here and for the HBB potential. The training data set for H_2_O and clusters from H_3_O^+^ to H_9_O_4_^+^ comprised 49,242 calculated energies, and the training and test sets for H_5_O_2_^+^ included 8869 and 711 energies, respectively. The RMSE was found to be 0.07 kJ/mol/atom for the training set and 0.09 kJ/mol/atom for the test set. These correspond to RMSE values of 53 and 41 cm^–1^, respectively, and can be compared to the values of 36 cm^–1^ for our (unweighted) PES110 K with 8717 coefficients and 13 cm^–1^ for our (unweighted) PES60K with 8001 coefficients. However, our PES110 K data set consists of 48,199 geometries/energies up to 110,046 cm^–1^, whereas their training and test sets consist of (a total of) 13,600 geometries/energies up to about 28,000 cm^–1^. Thus it would be more appropriate to compare their PES to our 8001 coefficient PES60K fit, which is based on 43523 geometries/energies extending up to 60,000 cm^–1^. Based on the above data, our fit to CCSD­(T) data appears to be about three times more precise than the BBSM fit to the CCSD­(T*)-F12a data. Using the CCSD­(T*)-F12a data as the target, ref 19 reports that the BBSM PES is about three times more accurate when compared to the HBB potential. These statements are not inconsistent because the less precise BBSM fit was trained on the more accurate CCSD­(T*)-F12a data, whereas our more precise fit and the HBB fit are fit to the less accurate CCSD­(T) data. While it would be instructive to compare directly the current surface with the BBSM one in terms of the values of *D*_e_ and the potential energy cuts for dissociation along the O–O distance, this information for the BBSM surface is not available.^19,25^ We note that their H_5_O_2_^+^ data set, from the SI of ref 25, includes only 29 points with O–O distances larger than 7 bohr, and the largest of these is 11.4 bohr, which on our O–O PES cut would be at only 86% of the dissociation energy. While longer distances from larger clusters were incorporated into the total learning, it seems unlikely that the BBSM surface would correctly describe dissociation of the H_5_O_2_^+^ Zundel cation. Of course, the objective of their study was to develop a general potential for the hydrated proton using the Behler–Parinello NN fitting method, in contrast to our approach, which is focused on the Zundel cation. It is worth noting that earlier Yu and Bowman developed a general hydrated proton potential, based on a many-body representation in which the Zundel cation is the 2-b hydronium–water interaction.^17,20^ Because this 2-b interaction can extend to large distances, accurate dissociation of the Zundel cation potential is important.

## Summary and Conclusions

We reported two new global PESs for the Zundel (H_5_O_2_^+^) cation using permutationally invariant polynomial fitting to a data set of energies up to 110,046 cm^–1^. Recommended surfaces for fits to energies up to 60,000 cm^–1^ and up to 110,046 cm^–1^ are given by PES60K and PES110K, respectively, as described above. Both PESs are also provided with fast reverse derivative gradients and are available for download. Diffusion Monte Carlo calculations, comparisons of stationary points, and calculation of the potential energy cut as a function of O–O distances show good agreement both with results on the HBB surface and with the CCSD­(*T*) results, where available.

## Data Availability

The full data set and the source code for the PES60K are available for download at https://github.com/jmbowma/QM-22. This is the faster program. Those interested in PES110k should contact the authors.
